# CtWRKY41 Transcription Factor from *Cynanchum thesioides* Mediates Salt Stress Resistance and Controls Flowering Time

**DOI:** 10.3390/plants14111716

**Published:** 2025-06-04

**Authors:** Xiaoyao Chang, Xiaoyan Zhang, Xiumei Huang, Fenglan Zhang, Zhongren Yang

**Affiliations:** 1 Inner Mongolia Key Laboratory of Wild Peculiar Vegetable Germplasm Resource and Germplasm Enhancement, College of Horticultural and Plant Protection, Inner Mongolia Agricultural University, Huhhot 010010, China; changxiaoyao0219@163.com (X.C.); zhangxiaoyan5329@163.com (X.Z.); 2 Department of Horticulture and Landscape Technology, Vocational and Technical College, Inner Mongolia Agricultural University, Baotou 014100, China; huangxm0404@126.com

**Keywords:** *Cynanchum thesioides*, CtWRKY41, gene expression and analysis, flowering time, salt stress

## Abstract

*Cynanchum thesioides* (Freyn) K. Schum is an ecologically significant species inhabiting the desert and semi-desert regions of northwestern China, distinguished by its remarkable resilience to environmental stressors. Elucidating the functional roles of its stress-responsive genes not only advances the theoretical framework of plant stress tolerance but also provides valuable genetic resources for stress-resilient crop breeding. This study identified a WRKY transcription factor, *CtWRKY41*, which is strongly induced by salt stress and plays a pivotal role in regulating both flowering time and abiotic stress responses. Subcellular localization analysis confirmed that *CtWRKY41* resides in the nucleus and exhibits transcriptional activation activity. *Arabidopsis* plants overexpressing *CtWRKY41* exhibited a significant delay in flowering and enhanced tolerance to salt stress. Further investigation revealed that *CtWRKY41* enhances stress resilience by markedly increasing antioxidant enzyme activity, promoting proline accumulation, and upregulating multiple stress-responsive genes. These coordinated mechanisms collectively contribute to the improved salt stress tolerance observed in transgenic *Arabidopsis*. This study underscores the regulatory significance of *CtWRKY41* in plant stress adaptation and establishes a theoretical basis for its potential application in crop genetic improvement programs aimed at enhancing stress resistance.

## 1. Introduction

An optimal soil environment is essential for nutrient acquisition and the sustained growth and development of crops. However, factors such as global climate change, rising sea levels, and unsustainable agricultural practices have exacerbated soil salinization, significantly impairing crop productivity and yield [1]. To withstand salt stress, plants have evolved intricate response and tolerance mechanisms over long-term adaptation, encompassing hormone signaling pathways [1], the Salt Overly Sensitive (SOS) signaling cascade [2], chromatin modifications [3], and transcriptional regulation [4].

As pivotal regulators of gene transcription, transcription factors play a fundamental role in signal transduction and gene expression modulation under environmental stress conditions [5]. These proteins recognize and bind to *cis*-regulatory elements in the promoter regions of target genes, orchestrating their transcriptional activity through activation or repression. Among them, the WRKY transcription factor family, exclusive to plants, comprises a diverse array of members with multifunctional roles. Beyond their involvement in plant growth and developmental processes, WRKY transcription factors are instrumental in mediating responses to both biotic and abiotic stressors [6,7]. Accumulating evidence highlights their critical role in salt stress tolerance, where they contribute to the establishment of salt-resistant phenotypes via complex regulatory networks [5].

All WRKY transcription factors share one or two highly conserved WRKY domains, each spanning approximately 60 amino acid residues. The N-terminal region of the WRKY domain harbors the highly conserved WRKYGQK motif, while the C-terminal region contains a characteristic zinc finger motif, classified into either the C2H2 (CX4-5CX22-23HX1H) or C2HC (C-X7-C-X23-HX1-C) type. These structural features collectively determine the DNA-binding specificity and regulatory function of WRKY proteins [8]. Based on the number of WRKY domains and the structural characteristics of their zinc finger motifs, WRKY transcription factors are categorized into three major groups [9]. WRKY transcription factors are classified into three major groups based on their structural characteristics. Group I WRKY proteins contain two WRKY domains, enabling them to bind a broader range of target genes compared to other groups, which provides significant advantages in complex signal integration and makes them particularly important in osmotic stress responses [4,5,9]. Group II WRKY proteins possess a single WRKY domain and a C_2_H_2_-type zinc finger motif and are further divided into five subgroups (IIa-IIe). This subgroup diversification results in functional specialization, allowing Group II WRKYs to play crucial roles in plant growth and development as well as in hormone-mediated responses to both biotic and abiotic stresses [9,10]. Group III WRKY proteins also contain a single WRKY domain but feature a distinctive C_2_HC-type zinc finger structure. This unique configuration often associates them with biotic stress responses, where they play key roles in plant defense against pathogens and other biological threats [8,11].

Recent studies have extensively demonstrated the pivotal role of WRKY transcription factors in plant responses to abiotic stress. For example, *TaWRKY17* enhances salt tolerance in both *Arabidopsis* and wheat [12], while *SlWRKY8* improves drought and salt resistance in tomatoes [13]. In contrast, *BnaA10.WRKY75* attenuates salt tolerance in *Arabidopsis* by promoting the accumulation of reactive oxygen species (ROS) [14]. Abscisic acid (ABA), a key phytohormone, is central to plant stress adaptation. Under abiotic stress, ABA biosynthesis is upregulated while its degradation is suppressed, leading to an increased endogenous ABA concentration [15]. Within an optimal concentration range, ABA induces stomatal closure, thereby mitigating water loss [16]. Signal transduction under abiotic stress operates through both ABA-dependent and ABA-independent pathways [17,18,19]. Several WRKY transcription factors have been implicated in abiotic stress responses via ABA signaling. For instance, *TaWRKY79* expression is induced by salt stress and ABA, and its overexpression in *Arabidopsis* confers increased salt tolerance by reducing ABA sensitivity, promoting primary root elongation under salt stress, and upregulating ABA-responsive genes [20]. Similarly, *GhWRKY6* enhances salt tolerance in *Arabidopsis* by scavenging ROS and modulating ABA signaling [21]. These findings underscore the diverse regulatory mechanisms by which WRKY transcription factors orchestrate plant stress responses.

WRKY transcription factors have also attracted considerable attention for their roles in the regulation of flowering time. Studies have shown that *AtWRKY71* promotes flowering in *Arabidopsis* by directly regulating FT expression [22], whereas overexpression of *IIWRKY22* and *RtWRKY23* in *Arabidopsis* delays flowering [23,24]. These findings underscore the integral role of WRKY transcription factors in the complex regulatory network governing flowering. Notably, certain WRKY transcription factors function at the intersection of stress responses and flowering regulation. For instance, *IIWRKY22* not only enhances salt tolerance in *Arabidopsis* but also modulates flowering time by regulating the expression of flowering-related genes [23]. Additionally, *WRKY71* counteracts salt-induced flowering delay in *Arabidopsis thaliana* [25]. This dual functionality likely represents an adaptive strategy evolved to prioritize survival over reproduction under adverse environmental conditions. However, the molecular mechanisms through which WRKY transcription factors integrate stress responses with developmental processes remain largely unresolved.

*Cynanchum thesioides* (Freyn) K. Schum., an erect semi-shrub of significant economic importance, is characterized by its robust adaptability and rapid growth. Predominantly distributed in arid and saline-alkali regions [26], it holds considerable medicinal, nutritional, and forage value. Moreover, its exceptional stress tolerance renders it an ideal candidate for ecological restoration and wasteland reclamation. To identify stress-resistant gene resources, a previous transcriptomic analysis revealed multiple WRKY transcription factors responsive to various environmental stresses [7]. Among these, *CtWRKY41* shows positive responsiveness to salt stress while exhibiting differential expression patterns across different floral bud developmental stages, suggesting its potential dual functionality in both stress adaptation and developmental regulation. In this study, *CtWRKY41* was successfully cloned, and its roles in salt stress tolerance and flowering regulation were systematically analyzed. These findings not only provide new insights into the functional diversity of the WRKY gene family but also establish a theoretical framework for leveraging *CtWRKY41* in crop stress resistance improvement strategies.

## 2. Materials and Methods

### 2.1. Plant Materials

The seeds of *C. thesioides* were sourced from Inner Mongolia Agricultural University, Huhhot, Inner Mongolia Province, Northwest China (111°69′ E, 40°80′ N). *Arabidopsis thaliana* (*Columbia*) seeds and the plant eukaryotic expression vector pCAMBIA1300-35S-EGFP were maintained in the laboratory.

### 2.2. Cultivation and Treatment of Cynanchum thesioides Seedlings

Seedlings (6-leaf stage) of *C. thesioides* were cultivated following the previously established protocol [26] and subsequently utilized in all experimental assays. Stress treatments included exposure to 10 μmol·L^−1^ ABA (0, 3, 6, 12, 24 h), 200 mmol·L^−1^ NaCl (0, 3, 6, 12, 24 h), and 20% PEG (0, 3, 6, 12, 24 h). Each treatment condition was applied to five seedlings, with three biological replicates. Meanwhile, floral buds of *C. thesioides* at different developmental stages were collected according to the method described by Chang et al. [27] for subsequent analyses. Post-treatment, plant materials were immediately collected, flash-frozen in liquid nitrogen, and stored at −80 °C for downstream analyses.

### 2.3. Quantitative Real-Time PCR (qRT-PCR) Analysis

RNA extraction and gene expression quantification were conducted according to previously described methodologies [27]. *ACT7* served as an internal reference gene [7]. Relative transcript levels of target genes were calculated using the 2^−ΔΔCT^ method, with all experiments performed using three biological replicates. Primer sequences used in this study are provided in Supplemental Table S1.

### 2.4. Detection of CtWRKY41 Subcellular Localization and Transcriptional Activation Assay

For subcellular localization analysis, the pCAMBIA1300-35S-eGFP vector was employed. The vector was digested with KpnI and Xba I, and the coding sequence (CDS) of *CtWRKY41* was inserted into the KpnI/Xba I site (TaKaRa, Dalian, China) via seamless cloning. Subcellular localization was assessed following a previously reported protocol [27]. The recombinant plasmids were transformed into *Agrobacterium tumefaciens* GV3101 competent cells. The bacterial suspension was mixed with the nuclear localization marker plasmid pBI221-NLS-CFPHyg at equal volume and concentration (OD_600_ = 0.8) and incubated at room temperature for 3 h to activate bacterial cells. The suspension was then infiltrated into young leaves of *Nicotiana benthamiana* using the syringe infiltration method. Inoculated plants were cultured in a growth chamber at 22 °C under light for 48 h. Samples were examined via confocal laser scanning microscopy. Three independent biological replicates were performed, with ≥5 leaf regions analyzed per replicate.

Additionally, the coding sequence (CDS) of *CtWRKY41* was cloned into the pGBKT7 vector to evaluate transcriptional activity. The recombinant plasmid, pGBKT7-CtWRKY41, was transformed into the *Saccharomyces cerevisiae* Y2H Gold strain (Coolaber, Beijing, China), while the empty pGBKT7 vector served as a negative control. Transformants were initially selected on SD/−Trp medium, followed by transfer to selective SD/−Trp/−His/−Ade medium, and incubated at 28 °C for three days to assess transcriptional activation.

### 2.5. Phylogenetic and Motif Analysis of CtWRKY41

For phylogenetic and motif analyses, homologous amino acid sequences of *CtWRKY41* from other species were retrieved via BLAST searches on the NCBI database. Multiple sequence alignment was performed using Clustal X (version 2.1) and a phylogenetic tree was constructed using the Neighbor-Joining method in MEGA 5.0. Conserved protein motifs were analyzed using MEME Suite (version 5.5.0).

### 2.6. Evaluation of Tolerance of INVSc1 to Salt Stress

The cloning vector and pYES2 vector were digested with EcoR I and Xba I (TaKaRa, Dalian, China) restriction enzymes, respectively. The target gene fragment and vector backbone were separated via agarose gel electrophoresis, excised, and purified. The purified target gene fragment was subsequently ligated into the linearized pYES2 vector to generate the overexpression construct pYES2-CtWRKY41. Both the empty pYES2 vector and the recombinant pYES2-CtWRKY41 construct were then transformed into *Saccharomyces cerevisiae* competent cells (INVSC1). Positive clones were screened and validated through sequencing.

For yeast culture and stress treatment, single colonies harboring either pYES2-CtWRKY41 or the empty pYES2 vector were inoculated into an SC-U liquid medium supplemented with 2% glucose and incubated at 30 °C for 16–20 h. The cultures were centrifuged at 5000 r/min for 1 min, and the resulting cell pellet was resuspended in 5 mL of SC-U induction medium containing 2% galactose, followed by a 24 h incubation at 30 °C to induce gene expression. After an additional centrifugation step, cells were resuspended in 0.9% NaCl solution, and the OD_600_ was adjusted to 0.8.

For the solid medium stress assay, SC-U solid selection medium supplemented with 2% glucose and either 0 M or 1 M NaCl was prepared. The yeast suspension was serially diluted 10-, 100-, 1000-, and 10,000-fold, and 2 μL of each dilution was spotted onto SC-U solid selection plates with varying salt concentrations. Plates were incubated at 30 °C for 2 days, and colony growth was assessed. Three biological replicates were included in each experiment.

For the liquid medium stress assay, the yeast suspension was adjusted to an OD_600_ of 0.2, and 1 mL of the suspension was centrifuged. The resulting pellet was resuspended in 1 mL of SC-U liquid selection medium containing 2% glucose, supplemented with different concentrations of NaCl. OD_600_ measurements were taken every 6 h over a 48 h period to monitor cell growth under salt stress conditions. All experiments were performed in triplicate.

### 2.7. Stable Arabidopsis Lines Overexpressing CtWRKY41

To further investigate the function of CtWRKY41 in plants, we constructed a 35S-CtWRKY41-eGFP recombinant vector using the pCAMBIA1300 backbone (harboring a hygromycin resistance marker, HYG) and introduced it into A. thaliana via Agrobacterium tumefaciens-mediated floral dip transformation. Inflorescences were immersed in the bacterial suspension during the bolting stage, and mature T_0_; seeds were collected, air-dried, and subjected to 4 °C vernalization for 3 days to synchronize germination. Transgenic T_0_; seedlings were screened on 1/2 MS medium supplemented with 50 mg/L HYG, and resistant plants were transferred to soil for growth. T_1_; and T_2_ generations were obtained by self-pollination, with each generation selected using the same HYG-containing medium to isolate homozygous lines. Genomic DNA from homozygous transgenic and wild-type plants was extracted for PCR verification using CtWRKY41-specific primers, confirming stable transgene integration.

Total RNA was then extracted from HYG-resistant, PCR-positive homozygous lines, and qRT-PCR analysis was performed to quantify *CtWRKY41* transcript levels, ensuring successful overexpression in transgenic lines. Mature T_3_ seeds were collected from validated homozygous T_2_ plants, air-dried, and stored at 4 °C for subsequent stress phenotype analyses. Seeds were surface-sterilized by sequential treatment with 70% ethanol for 10 min and absolute ethanol for 20 min before being sown onto 1/2 MS solid medium. Following a 2-day stratification period at 4 °C, the seeds were transferred to a growth chamber maintained at 24 °C under a 16 h light/8 h dark photoperiod with 70% relative humidity. Once seedlings developed approximately four true leaves, they were transplanted into nutrient soil for further cultivation. Salt stress treatment was initiated 14 days after transplantation by irrigating the plants with 100 mL of 150 mmol·L^−1^ NaCl solution every 3 days. After 14 days of stress treatment, the whole-plant fresh weight was recorded, and leaf samples (4th to 6th rosette leaves from the base) were collected for subsequent analysis. For germination and root length assays, *Arabidopsis* seeds were sown onto 1/2 MS solid medium (10 cm × 10 cm square plates) supplemented with 150 mmol·L^−1^ NaCl and 0.6 μM ABA following a 2-day vernalization period. Germination rates were recorded every 12 h, while root length was measured after 14 days using a digital vernier caliper.

In salt-stressed *A. thaliana* plants, quantitative real-time PCR (qRT-PCR) analysis was performed using gene-specific primers (*AtCAT1*, *AtPOD1*, *AtAPX1*, *AtNCED3*, *AtABI1*, *AtABI2*, *AtSOS1*, and *AtRD22*), with *A. thaliana* Actin1 serving as the reference gene. Relative expression levels of target genes were calculated using the 2^−ΔΔCt^ method. The experiment included three biological replicates with three technical replicates each to ensure data reliability.

### 2.8. Phenotypic Observation of Arabidopsis thaliana and Determination of Related Physiological and Biochemical Indexes

Phenotypic parameters, including bolting time, flowering time, and the number of rosette leaves, were monitored daily. Flowering time was defined as the period from sowing to the full opening of the first flower. The expression profiles of flowering- and stress-responsive genes in *Arabidopsis* seedlings were assessed via qRT-PCR. Oxidative stress markers, including malondialdehyde (MDA), superoxide anion (O_2_^−^), and hydrogen peroxide (H_2_O_2_) levels, as well as the enzymatic activities of superoxide dismutase (SOD), peroxidase (POD), and catalase (CAT), were quantified following the manufacturer’s protocol of the respective assay kits (Solarbio, Beijing, China). Diaminobenzidine (DAB) and nitrotetrazolium blue chloride (NBT) staining were performed according to previously established protocols [27].

### 2.9. Statistical Analysis

Statistical analyses were conducted using SPSS Statistics version 26.0 (IBM, Armonk, NY, USA) to evaluate treatment differences. One-way ANOVA was employed for multiple group comparisons, followed by Student’s *t*-test for pairwise comparisons. Additionally, Pearson’s correlation analysis was performed to assess linear relationships between variables. Significance levels were denoted as follows: ** *p* < 0.01.

## 3. Results

### 3.1. Sequence Characteristics and Expression Patterns of CtWRKY41

**Figure 1:**
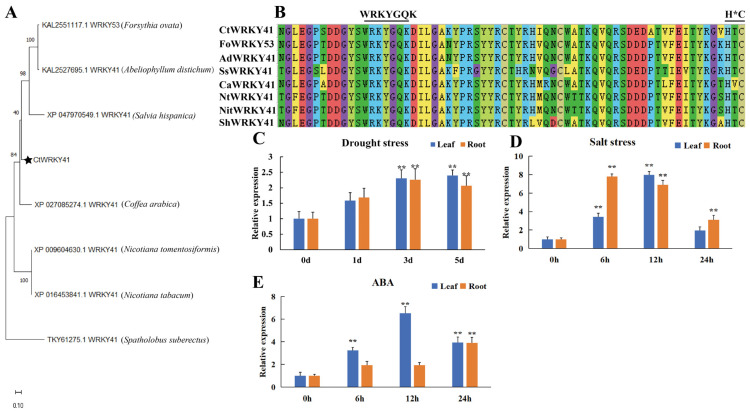
Multiple sequence alignment, phylogenetic tree, and expression pattern analysis of *CtWRKY41*. (**A**) Phylogenetic relationship between *CtWRKY41* and WRKY proteins from other plants. The portion highlighted with a pentacle is *CtWRKY41*, (**B**) alignment of the amino acid sequences of *CtWRKY41* and WRKY from other plants, (**C**) *CtWRKY41* expression level under drought stress, (**D**) shows the *CtWRKY41* expression level under salt stress, (**E**) represents the *CtWRKY41* expression level under ABA stress. All data represent the mean ± standard error of three independent experiments, and asterisk indicates a statistically significant difference between the expression levels of each gene in the treated samples compared to the wild-type (WT) control (Student’s *t*-test; ** *p* < 0.01). WRKYGQK: Represents the highly conserved heptapeptide sequence in WRKY transcription factors. H*C: Represents the zinc finger motif. In the sequence alignment, amino acids of the same color in each column indicate conserved amino acid residues with identical properties.

A comprehensive analysis of the WRKY gene family previously identified a WRKY transcription factor that is significantly upregulated under salt stress [7]. This gene, subsequently cloned and designated *CtWRKY41*, contains an open reading frame (ORF) of 1080 bp, encoding a 359-amino acid protein (Figure S1). Sequence alignment analysis revealed that *CtWRKY41* shares high sequence homology with WRKY proteins from multiple species, including *Forsythia ovata WRKY53* (KAL2551117.1), *Abeliophyllum distichum WRKY41* (KAL2527695.1), and *Salvia hispanica WRKY41* (XP_047970549.1) (Figure 1A). Phylogenetic analysis further confirmed that *CtWRKY41* contains the characteristic N-terminal WRKYGQK motif and C-terminal C_2_HC zinc finger domain (Figure 1B). The presence of these conserved domains, supported by multispecies sequence comparisons, classifies *CtWRKY41* as a member of the Group III WRKY transcription factor family (Figure S2).

The tissue-specific expression pattern of *CtWRKY41* in *C. thesioides* was examined using qRT-PCR. Abiotic stress treatment assays revealed that *CtWRKY41* expression was markedly induced by both NaCl and drought stress, with the most pronounced upregulation observed under salt stress conditions (Figure 1C,D). Additionally, ABA treatment significantly elevated *CtWRKY41* expression after 6 h (Figure 1E), suggesting its potential regulation via the ABA signaling pathway. These results indicate that *CtWRKY41* plays a critical role in the salt stress response of *C. thesioides*.

### 3.2. Subcellular Localization and Transcriptional Activation of CtWRKY41

**Figure 2:**
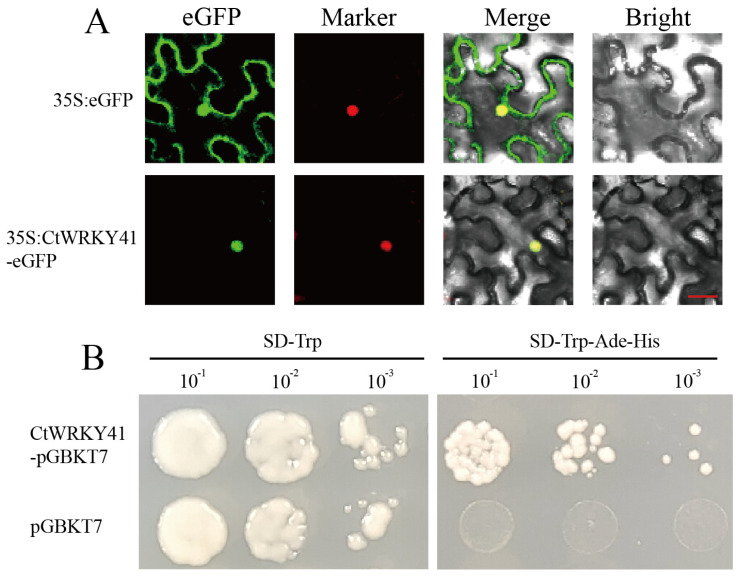
Subcellular localization and transcriptional activation of *CtWRKY41*. (**A**) Subcellular localization of *CtWRKY41* protein in leaves of Nicotiana benthamiana. From left to right: green fluorescent protein (eGFP), Marker: pBI221-NLS-CFP (nucleus), merged, and bright-field. Bars  =  20 μm. (**B**) Transcriptional activation of *CtWRKY41* in yeast cells.

To determine the subcellular localization of *CtWRKY41*, a fusion construct encoding *CtWRKY41* tagged with a green fluorescent protein (GFP) was generated and co-expressed with the nuclear marker in *N. benthamiana* epidermal cells. Confocal microscopy analysis showed that the CtWRKY41-GFP fluorescence signal was exclusively localized to the nucleus, whereas the control GFP signal was distributed in both the cytoplasm and nucleus (Figure 2A), confirming the nuclear localization of *CtWRKY41*.

To assess its transcriptional activation potential, a yeast one-hybrid assay was performed. The recombinant plasmid CtWRKY41-pGBKT7 and the empty pGBKT7 vector (negative control) were transformed into *Saccharomyces cerevisiae* Y2Hgold cells. Yeast harboring either construct exhibited growth on synthetic dropout medium lacking tryptophan (SD/−Trp). However, only yeast cells expressing CtWRKY41-pGBKT7 were able to grow on a selective medium lacking tryptophan, histidine, and adenine (SD/−Trp-His-Ade) (Figure 2B), demonstrating that *CtWRKY41* possesses transcriptional activation activity. Collectively, these results establish *CtWRKY41* as a nuclear-localized transcription factor.

### 3.3. Overexpression CtWRKY41 Enhanced Yeast Resistance to NaCl

**Figure 3:**
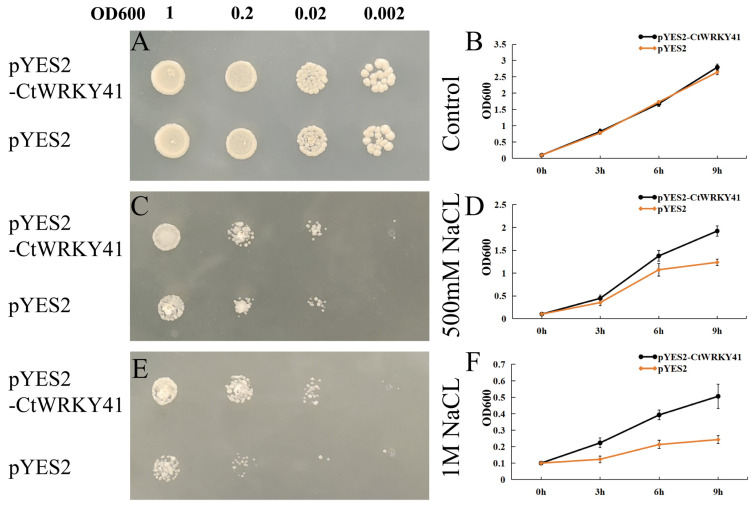
Functional analysis of the *CtWRKY41* gene in yeast. Growth phenotypes of transgenic yeast on solid medium supplemented with (**A**) 0 mM, (**C**) 500 mM, and (**E**) 1 M NaCl. Growth status of transgenic yeast in liquid medium supplemented with (**B**) 0 mM, (**D**) 500 mM, and (**F**) 1 M NaCl.

The role of *CtWRKY41* in yeast growth and stress resistance was evaluated in *S. romyces cerevisiae* strains harboring the CtWRKY41-overexpression construct (pYES2-CtWRKY41). Under standard growth conditions, *CtWRKY41* overexpression did not significantly impact yeast proliferation (Figure 3A). However, under salt stress, yeast cells expressing *CtWRKY41* exhibited markedly enhanced resistance on a solid medium containing NaCl (500 mM and 1 M), particularly at a 1000-fold dilution (Figure 3C,E). In liquid culture assays, the growth kinetics of CtWRKY41-overexpressing yeast and control strains were comparable under non-stress conditions (Figure 3B). However, upon exposure to increasing NaCl concentrations, the survival rate of the CtWRKY41-overexpressing strain consistently exceeded that of the control. Notably, under 500 mM and 1 M NaCl stress, the survival rate of the overexpressing yeast strain remained significantly higher than that of the empty vector control after 9 h of treatment (Figure 3D,F). These results suggest that *CtWRKY41* enhances yeast salt tolerance.

### 3.4. CtWRKY41 Participates in ABA Response and Enhances Salt Tolerance in Arabidopsis

To further investigate the function of CtWRKY41, Arabidopsis transformation was performed using the floral dip method. Positive transgenic seedlings were selected (Figure S3) and verified through genomic PCR (Figure S4) and qRT-PCR quantification of CtWRKY41 transcripts (Figure S5) to obtain homozygous lines.

**Figure 4:**
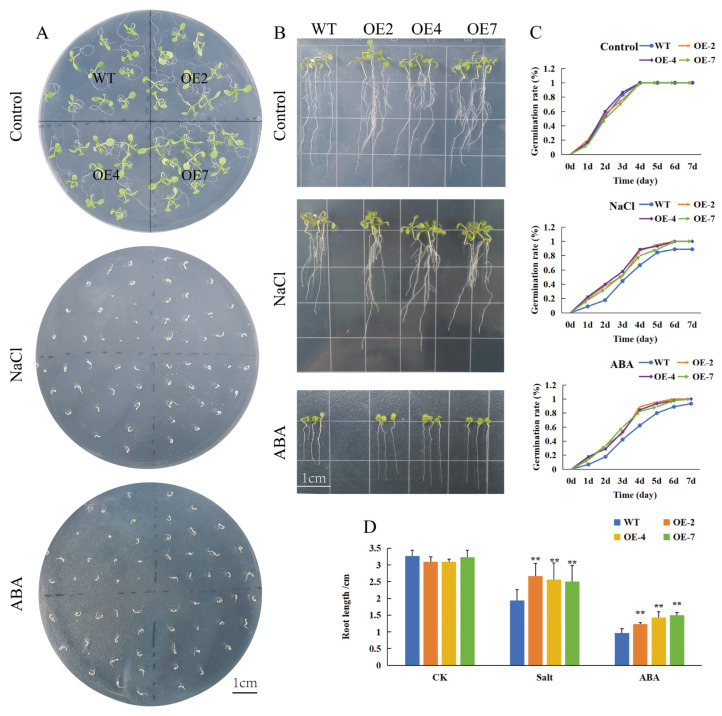
Overexpression of *CtWRKY41* enhances salt tolerance and reduces ABA sensitivity in Arabidopsis seeds. (**A**) Germination phenotypes under different stress conditions (7 days after germination). (**B**) Root length growth phenotypes under different stress conditions, (**C**) root length, and (**D**) germination rate of wild-type (WT) and transgenic Arabidopsis lines grown on 1/2 MS medium supplemented with 0.6 μM ABA and 200 mmol/L NaCl. All data represent the mean ± standard error of three independent experiments, and asterisk indicates a statistically significant difference between the expression levels of each gene in the treated samples compared to the wild-type (WT) control (Student’s *t*-test). Values marked with two star were considered significant at *p* < 0.01.

To determine whether *CtWRKY41* is involved in the ABA signaling pathway, the sensitivity of *CtWRKY41* transgenic lines to ABA treatment was assessed through germination assays. Exposure to 0.6 μM ABA significantly reduced germination rates in both CtWRKY41-overexpressing (OE) lines and wild-type (WT) plants; however, the inhibition was more pronounced in WT plants (Figure 4A,D). Under salt stress conditions, NaCl treatment delayed germination and reduced germination rates in both genotypes, yet transgenic plants exhibited significantly higher germination rates than WT. To further evaluate the role of *CtWRKY41* in ABA and salt stress responses, root elongation assays were performed. Under normal growth conditions, *CtWRKY41* overexpression promoted lateral root development in OE lines. Following a 7-day treatment with 0.6 μM ABA, the primary root length of transgenic lines remained significantly longer than that of WT plants. Similarly, under 150 mM NaCl stress, root growth was inhibited in both genotypes; however, the suppression was significantly less severe in OE lines compared to WT (Figure 4B,C). These results suggest that *CtWRKY41* overexpression reduces *Arabidopsis* sensitivity to exogenous ABA while enhancing salt stress tolerance. This indicates that *CtWRKY41* may function as a key regulator integrating ABA signaling and salt stress responses.

### 3.5. CtWRKY41 Enhances Physiological Indicators and Antioxidant Capacity in Transgenic Arabidopsis

**Figure 5:**
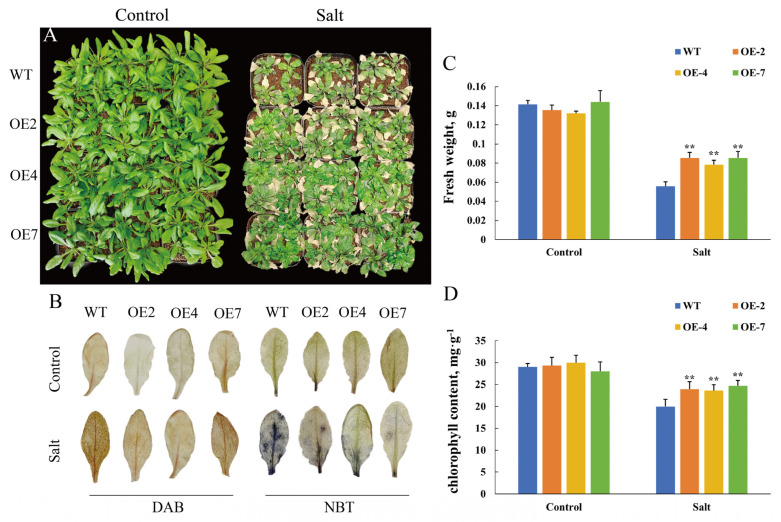
Phenotypic analysis, fresh weight, and chlorophyll content of A. thaliana under salt stress. (**A**) Growth phenotype of A. thaliana under salt stress conditions. (**B**) Histochemical staining using DAB (brown) and NBT (blue) to detect reactive oxygen species (ROS) accumulation. (**C**) Fresh weight and (**D**) total chlorophyll content in leaves of A. thaliana following salt stress treatment. All data represent the mean ± standard error of three independent experiments, and asterisk indicates a statistically significant difference between the expression levels of each gene in the treated samples compared to the wild-type (WT) control (Student’s *t*-test). Values marked with two star were considered significant at *p* < 0.01.

To comprehensively assess the role of *CtWRKY41* in salt tolerance, transgenic and WT *A. thaliana* plants cultivated in nutrient soil were subjected to salt stress treatment. Under normal growth conditions, no significant differences in growth status or phenotype were observed between CtWRKY41-OE lines and WT plants. However, following salt stress treatment, both genotypes exhibited growth inhibition characterized by stunted development and leaf dehydration, with OE lines displaying significantly less severe symptoms, including reduced chlorosis and wilting (Figure 5A). ROS accumulation was assessed via nitrotetrazolium blue chloride (NBT, blue) and diaminobenzidine (DAB, brown) staining. Under non-stress conditions, leaf staining patterns were comparable between WT and OE lines. However, under salt stress, OE plants exhibited markedly lower levels of blue and brown precipitate compared to WT (Figure 5B), indicating reduced accumulation of H_2_O_2_ and O_2_^–^. Furthermore, the fresh weight and chlorophyll content of OE lines remained significantly higher than those of WT plants (Figure 5C,D). These results collectively demonstrate that *CtWRKY41* enhances salt tolerance in *Arabidopsis* by improving antioxidant capacity and maintaining photosynthetic efficiency.

**Figure 6:**
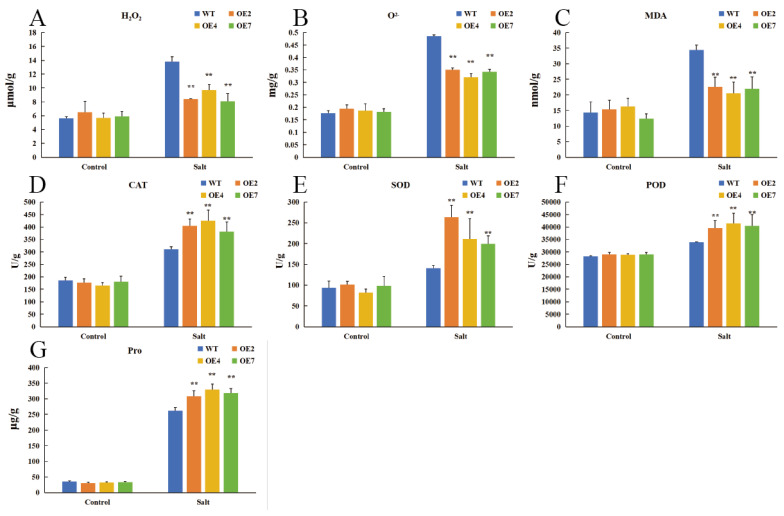
Assessment of oxidative damage and antioxidant enzyme activity in *A. thaliana* under salt stress. (**A**) Hydrogen peroxide (H_2_O_2_) content; (**B**) Superoxide anion (O^2−^) content; (**C**) Malondialdehyde (MDA) content; (**D**) Catalase (CAT) activity; (**E**) Superoxide dismutase (SOD) activity; (**F**) Peroxidase (POD) activity; (**G**) Proline (Pro) content. All data represent the mean ± standard error of three independent experiments, and asterisk indicates a statistically significant difference between the expression levels of each gene in the treated samples compared to the wild-type (WT) control (Student’s *t*-test). Values marked with two star were considered significant at *p* < 0.01.

Salt stress typically induces excessive ROS accumulation in plants, leading to oxidative damage. In this study, salt stress significantly increased MDA, O_2_^−^, and H_2_O_2_ levels in *Arabidopsis* leaves. However, compared to WT plants, OE lines exhibited significantly lower MDA and H_2_O_2_ accumulation (Figure 6B,C). To further elucidate the antioxidant mechanisms mediated by *CtWRKY41*, antioxidant enzyme activities and osmoregulatory substance levels were quantified before and after salt stress exposure. Salt stress induced a substantial increase in the activities of SOD, CAT, and POD, as well as proline accumulation, in both WT and OE lines. Notably, the enzymatic activities and proline content were significantly higher in OE lines than in WT (Figure 6D–G). These results indicate that *CtWRKY41* mitigates ROS accumulation under salt stress by enhancing antioxidant enzyme activity and osmoregulatory capacity, thereby alleviating oxidative damage and conferring increased salt tolerance.

### 3.6. CtWRKY41 Regulates the Expression of Stress-Responsive Genes

**Figure 7:**
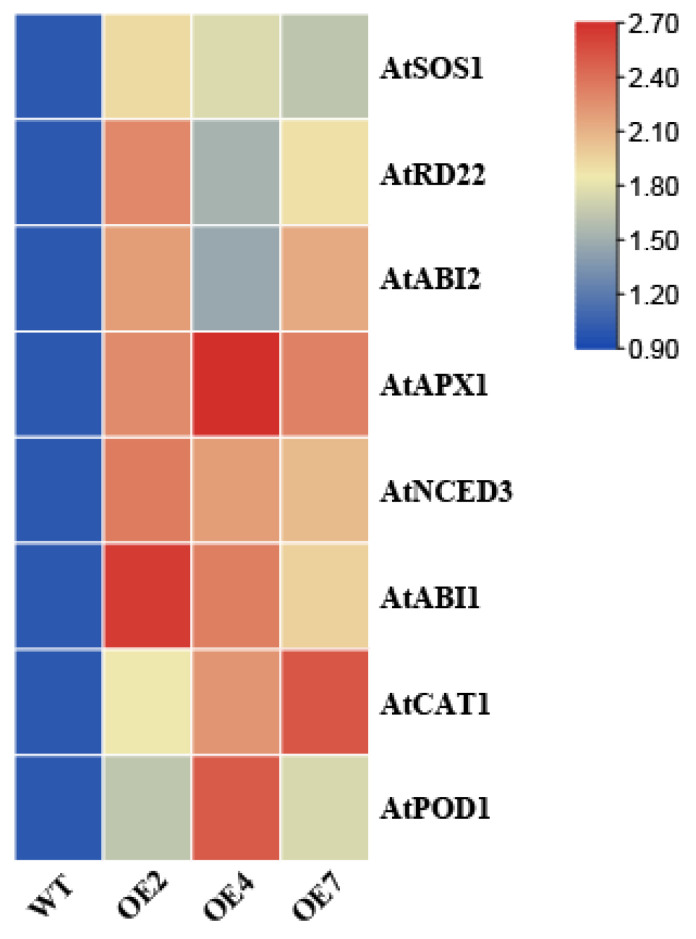
Expression of related genes under salt stress.

To elucidate the molecular mechanisms underlying CtWRKY41-mediated salt tolerance in *Arabidopsis*, the expression profiles of stress-responsive genes were examined in WT and CtWRKY41-OE lines under salt stress conditions. The results revealed that salt stress significantly upregulated the expression of multiple key genes in OE lines. Notably, genes encoding antioxidant enzymes, including *AtCAT1*, *AtPOD1*, and *AtAPX1*, exhibited markedly higher expression levels in OE lines compared to WT, indicating enhanced antioxidative capacity. Additionally, genes associated with the ABA signaling pathway, such as *AtNCED3*, *AtABI1*, and *AtABI2*, were significantly upregulated in OE lines, suggesting that *CtWRKY41* may modulate ABA-mediated stress responses. Furthermore, genes involved in ion homeostasis and osmotic stress tolerance, including the plasma membrane Na⁺ antiporter gene *AtSOS1* and the dehydration-responsive gene *AtRD22*, were also expressed at significantly higher levels in OE lines than in WT (Figure 7). These results indicate that *CtWRKY41* enhances *Arabidopsis* stress tolerance by coordinately regulating the expression of genes involved in antioxidant defense, ion transport, and ABA-dependent signaling pathways. This highlights its multifaceted role in modulating plant responses to abiotic stress through coordinated molecular mechanisms.

### 3.7. CtWRKY41 Delays Flowering Time in Transgenic Arabidopsis

**Figure 8:**
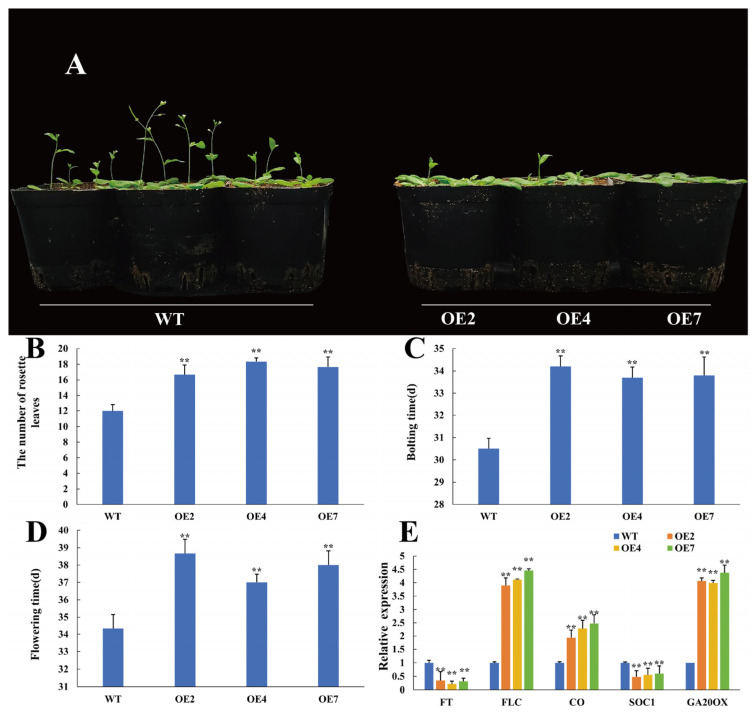
Phenotypic analysis of transgenic A. thaliana overexpressing *CtWRKY41*. (**A**) Phenotypic observation of transgenic A. thaliana overexpressing *CtWRKY41*. Scale bar = 2 cm. (**B**) Number of rosette leaves at the time of flowering. (**C**) Flowering time (defined as the opening of the first flower). (**D**) Bolting time (defined as the emergence of an inflorescence stalk of 1 cm in height). (**E**) Expression levels of endogenous flowering time-related genes in transgenic A. thaliana overexpressing *CtWRKY41*. All data represent the mean ± standard error of three independent experiments, and asterisk indicates a statistically significant difference between the expression levels of each gene in the treated samples compared to the wild-type (WT) control (Student’s *t*-test). WT: wild-type plants; OE-2, OE-4, and OE-7: independent *CtWRKY41* overexpression lines. Values marked with two star were considered significant at *p* < 0.01.

The differential expression of CtWRKY41 at different developmental stages of *Cynanchum thesioides* floral buds suggests that it may be involved in the regulation of flowering time (Figure S6). To investigate this hypothesis, a systematic analysis was conducted to assess the effects of *CtWRKY41* on floral transition. Seeds from WT and CtWRKY41-OE lines were vernalized and cultivated under short-day conditions. Flowering time was determined based on floral bud emergence, and the number of rosette leaves was recorded as an additional metric. The results (Figure 8A–D) revealed that WT plants bolted at an average of 30.5 days, with flowering occurring at 34.3 days and a rosette leaf count of 12. In contrast, CtWRKY41-OE lines exhibited a significant delay in bolting (34 days) and flowering (37.8 days), accompanied by an increase in rosette leaf number to 17.5. These results indicate that *CtWRKY41* overexpression markedly postpones flowering in *Arabidopsis*, suggesting a critical role in integrating environmental cues to modulate flowering time.

To further elucidate the molecular mechanism of CtWRKY41-mediated flowering regulation, qRT-PCR was employed to quantify the expression levels of key flowering-related genes in WT and CtWRKY41-OE lines (Figure 8E). The results demonstrated a significant downregulation of the floral integrator gene FT in CtWRKY41-OE plants, while the floral repressor *FLC* was markedly upregulated. Moreover, genes associated with the photoperiod pathway (CO) and gibberellin (GA) biosynthesis (GA20OX) exhibited significantly increased expression, whereas the floral integrator *SOC1* was notably suppressed in CtWRKY41-OE lines. These results suggest that *CtWRKY41* orchestrates flowering time through a complex regulatory network. By upregulating *FLC* and downregulating *FT* and *SOC1*, *CtWRKY41* suppresses the floral transition. Additionally, during the transition phase, *CtWRKY41* modulates the photoperiod and GA signaling pathways by promoting *CO* and *GA20OX* expression. This multifaceted regulatory framework underscores the pivotal role of *CtWRKY41* in coordinating multiple flowering-related pathways, ultimately resulting in a delayed flowering phenotype. These insights provide a deeper understanding of the involvement of WRKY transcription factors in the intricate genetic network governing floral transition in plants.

## 4. Discussion

As a key ecological species thriving in the arid and semi-arid regions of northwestern China, *C. thesioides* exhibits exceptional adaptability to harsh environmental conditions [7]. Understanding the mechanisms underlying its stress tolerance holds significant potential for the conservation and utilization of this unique plant species. In the present study, the *CtWRKY41* gene was successfully cloned, and stable transgenic *A. thaliana* lines overexpressing this gene were generated. Phenotypic and physiological analyses of these transgenic lines under salt stress demonstrated that *CtWRKY41* overexpression markedly enhanced salt tolerance in *Arabidopsis*.

Salt stress imposes severe constraints on plant growth and development, primarily by impairing transpiration, thereby limiting water and nutrient uptake. Additionally, salt-induced cytotoxicity disrupts key metabolic pathways, including photosynthesis and respiration, leading to the excessive accumulation of ROS [28,29,30]. To mitigate oxidative damage, plants typically enhance antioxidant enzyme activity and accumulate protective metabolites through transcriptional and metabolic regulation [31,32]. Chlorophyll, the primary pigment involved in plant photosynthesis, directly influences the photosynthetic efficiency of plants [33]. In this study, *CtWRKY41* overexpression significantly alleviated salt stress-induced growth inhibition in transgenic *Arabidopsis*, as evidenced by increased root length, fresh weight, and chlorophyll content. These findings suggest that *CtWRKY41* promotes photosynthesis, enhances water absorption, and supports root development under salt stress, thereby contributing to plant resilience in adverse environments.

Further investigation revealed that *CtWRKY41* overexpression effectively reduced ROS accumulation and oxidative damage in transgenic *Arabidopsis* leaves. This was accompanied by a significant upregulation of antioxidant enzyme-related genes (*AtSOD1*, *AtPOD1*, and *AtAPX1*) and an increase in the activities of SOD, CAT, and POD. These findings indicate that *CtWRKY41* enhances ROS-scavenging capacity by activating the transcription and enzymatic activity of components within the antioxidant defense system. Additionally, *CtWRKY41* overexpression promoted the biosynthesis and accumulation of proline, a critical osmoprotectant and antioxidant that plays a key role in maintaining cellular osmotic balance and neutralizing oxidative stress [31,32,34]. Collectively, these results demonstrate that *CtWRKY41* enhances plant stress resistance through a coordinated mechanism involving the activation of antioxidant defenses, accumulation of osmoprotectants, and maintenance of photosynthetic efficiency and root development.

Recent studies have increasingly demonstrated that WRKY transcription factors (TFs) are integral to plant responses to abiotic stress, primarily through their involvement in the ABA signaling pathway [35,36,37]. In plants, ABA accumulates via the ABA-dependent pathway, leading to the inhibition of seed germination, seedling growth, and overall development under stress conditions [34]. Notably, ABA treatment induced a significant upregulation of *CtWRKY41* expression (Figure 1E), suggesting its potential role in ABA-mediated stress responses. To further explore the function of *CtWRKY41* in salt stress adaptation and ABA signaling, the expression profiles of several stress-related marker genes, which play pivotal roles in plant defense against environmental challenges, were analyzed [38]. The results revealed varying degrees of upregulation in these genes (Figure 7). In CtWRKY41-overexpressing lines, two key ABA signaling regulators, *ABI1* and *ABI2*, exhibited significant transcriptional upregulation. As core components of ABA signal transduction, *ABI1* and *ABI2* are known to function as negative regulators of ABA responses [39]. Their elevated expression in CtWRKY41-OE lines was consistent with the observed ABA-insensitive phenotype during seed germination and seedling development (Figure 3A). Previous studies have indicated that increased expression of *ABI1* and *ABI2* can attenuate plant stress tolerance, whereas their downregulation enhances stress resilience [40,41]. However, this relationship is not absolute. For instance, overexpression of *GmbZIP44*, *GmbZIP62*, and *GmbZIP78* in *Arabidopsis* reduces ABA sensitivity while simultaneously enhancing abiotic stress tolerance, likely via upregulation of *ABI1* and *ABI2* within the ABA signaling cascade [42]. Conversely, overexpression of ZmPP2C suppresses *ABI1* and *ABI2* expression but also diminishes drought stress tolerance [43]. Additionally, transgenic *Arabidopsis* lines overexpressing *SmWRKY40* exhibit enhanced tolerance to both ABA and salt stress [38]. Plant stress tolerance is determined by a complex interplay between physiological adaptations and the expression of stress-responsive genes, with multiple regulatory factors contributing to enhanced resilience. These findings underscore the multifaceted role of WRKY transcription factors in modulating ABA signaling and stress responses, offering valuable insights into the molecular mechanisms underlying plant abiotic stress resistance.

Plants have evolved an intricate flowering regulation network to adapt to diverse environmental conditions. Within the photoperiod pathway, flowering is controlled by the derepression of *CO* and *FT* [44], while in the vernalization, temperature, and autonomous pathways, flowering is modulated through the release of *FLC*-mediated suppression of *FT* [45]. In the GA pathway, *SOC1* functions as a key floral integrator [46]. To elucidate the molecular mechanisms underlying *CtWRKY41*-mediated regulation of flowering, quantitative analysis was performed on the expression levels of five key genes across multiple flowering pathways (Figure 7). The results revealed a significant upregulation of *CO* and *GA20OX* in transgenic plants, suggesting that *CtWRKY41* primarily modulates flowering through the photoperiod and GA pathways. Concurrently, the upregulation of *FLC* and the downregulation of *FT* and *SOC1* further indicate that *CtWRKY41* delays flowering by repressing the expression of floral-promoting genes. These findings collectively demonstrate that *CtWRKY41* serves as a central regulatory factor integrating multiple flowering-related signaling pathways.

This study successfully cloned *CtWRKY41* from *C. thesioides*, a desert-adapted plant native to northwestern China, and functionally validated its role through transgenic expression in *A. thaliana*. The results revealed that *CtWRKY41* is functionally linked to the ABA signaling pathway and enhances the plant’s ROS scavenging capacity, mitigating oxidative damage induced by salt stress and improving overall stress tolerance. Notably, *CtWRKY41* exhibited a dual phenotypic effect: enhancing salt stress resistance while delaying flowering. This suggests that *CtWRKY41* may optimize plant survival strategies by integrating stress responses with developmental regulation. Under salt stress conditions, *CtWRKY41* promotes plant survival by increasing salt tolerance while simultaneously delaying flowering, thereby extending the vegetative growth phase and allowing plants to allocate more resources toward stress adaptation. This regulatory mechanism reflects an evolutionary strategy favoring survival over reproduction under adverse environmental conditions. These findings provide novel insights into the molecular basis of plant stress tolerance and developmental regulation while offering a theoretical framework for breeding crops with enhanced stress resistance and optimized flowering timing.

## 5. Conclusions

*CtWRKY41* exhibits a dual function in regulating flowering time and enhancing salt stress tolerance. Localized in the nucleus, it possesses transcriptional activation activity. Overexpression of *CtWRKY41* in *Arabidopsis* resulted in a significant delay in flowering time and improved resistance to salt stress. Mechanistically, *CtWRKY41* enhances stress tolerance by upregulating antioxidant enzyme activity, promoting the accumulation of the osmoprotectant proline, and inducing the expression of multiple stress-responsive genes. These findings underscore the multifaceted role of *CtWRKY41* in coordinating plant developmental processes and stress adaptation.

## Data Availability

Data is contained within the article or Supplementary Material.

## Supplementary Materials

The following supporting information can be downloaded at: https://www.mdpi.com/article/10.3390/plants14111716/s1, Figure S1: Cloning of CtWRKY53 gene; Figure S2: Phylogenetic relationship between CtWRKY41 and WRKY proteins from other plants. The portion highlighted with a pentacle is CtWRKY41; Figure S3: Screening of transgenic Arabidopsis-positive plants. The red arrow indicates the positive transgenic line; Figure S4: Note: M: DNA ladder marker 2000; +: PCR amplification of CtWRKY41 in Cynanchum thesioides; OE1-7: CtWRKY41 transgenic lines; WT: Wild-type A. thaliana; Figure S5: CtWRKY41 expression levels of wild type (WT), and transgenic lines (OE1, OE2, OE4, OE6, and OE7). Figure S6: *CtWRKY41* genes express at different developmental stages of the anther. Table S1: Primers used in this study.
