# Supplementary material for: CtWRKY41 Transcription Factor from Cynanchum thesioides Mediates Salt Stress Resistance and Controls Flowering Time
Source: Plants (Basel). 2025 Jun 4;14(11):1716. doi: 10.3390/plants14111716 (PMC12158058; doi:10.3390/plants14111716)
Supplement: Supplementary file 1 [file plants-14-01716-s001.zip › plants-3562120-supplementary.pdf]

```
        10         20         30         40         50         60         70         80         90
ATGAATTGTGCTACTAGCTGGGAATACAAGTCACTAATCAATGAGCTCACCCAAGGAATGGAGAAAGCTAAGCAGCTAAGAGTTCATTTG
 M  N  C  A  T  S  W  E  Y  K  S  L  I  N  E  L  T  Q  G  M  E  K  A  K  Q  L  R  V  H  L

       100        110        120        130        140        150        160        170        180
AGCTCAACTTCTTCGGATCAAGAGTTTCTGCTGCAGAGGATACTGTCTTCCTATGAGAAAGCTCTGTTGATCCTCAAGTGGAGTGGTTCC
 S  S  T  S  S  D  Q  E  F  L  L  Q  R  I  L  S  S  Y  E  K  A  L  L  I  L  K  W  S  G  S

       190        200        210        220        230        240        250        260        270
TCCACAGGACAATCGCAGCAGCCTACGCCACCAGGTTCTGCCTGTGGTGCCTTAGAGTCTTCTATTTCCGTGGACGGTAGCCCTCGCAGT
 S  T  G  Q  S  Q  Q  P  T  P  P  G  S  A  C  G  A  L  E  S  S  I  S  V  D  G  S  P  R  S

       280        290        300        310        320        330        340        350        360
GAGGAGCTAAACAGAAATTTCAGGGATCAGCAGGACCAGAACATGAATGCTTCCAAGAAGAGAAAGACATTGCCTACATGGACAGAACAA
 E  E  L  N  R  N  F  R  D  Q  Q  D  Q  N  M  N  A  S  K  K  R  K  T  L  P  T  W  T  E  Q

       370        380        390        400        410        420        430        440        450
GTGAAAGTCAGCTCTGACAATGGACTTGAAGGACCTTCTGATGATGGCTATAGTTGGAGAAAGTATGGACAGAAAGATATCTTGGGAGCC
 V  K  V  S  S  D  N  G  L  E  G  P  S  D  D  G  Y  S  W  R  K  Y  G  Q  K  D  I  L  G  A

       460        470        480        490        500        510        520        530        540
AAATATCCCAGGAGCTATTACAGATGCACGTACCGTCACATTCAAAACTGTTGGGCGACAAAGCAAGTTCAAAGATCTGATGAAGATGCC
 K  Y  P  R  S  Y  Y  R  C  T  Y  R  H  I  Q  N  C  W  A  T  K  Q  V  Q  R  S  D  E  D  A

       550        560        570        580        590        600        610        620        630
ACTGTATTTGAGATTACATACAGAGGAGTTCATACATGCAACCTTGCCGGAACCAGCAATTCAGTTGCTTCAACAGCATCTCCTGAAAAG
 T  V  F  E  I  T  Y  R  G  V  H  T  C  N  L  A  G  T  S  N  S  V  A  S  T  A  S  P  E  K

       640        650        660        670        680        690        700        710        720
CAAGAAGTGAGACATAAGAATAATTGCAGTAGCAACTATCAATTGCAGCAACCTAATCAGATGCTAATGAACCTTAGAGCAAACTTGCGA
 Q  E  V  R  H  K  N  N  C  S  S  N  Y  Q  L  Q  Q  P  N  Q  M  L  M  N  L  R  A  N  L  R

       730        740        750        760        770        780        790        800        810
GTTAATACTGGGGATTCAGAAAATAAGAACAGCACCTCCCCGTTTTCTTTCCCTTCAACACTAACATATTTTGAAGATGAAAATCAATAT
 V  N  T  G  D  S  E  N  K  N  S  T  S  P  F  S  F  P  S  T  L  T  Y  F  E  D  E  N  Q  Y

       820        830        840        850        860        870        880        890        900
TTCCCCATCTCAACACATGTTGATGAAAATCTCATGGGGACTTATTCACCATCCTTCATATCACCAGCAACATCTGAATCAAACTACTTT
 F  P  I  S  T  H  V  D  E  N  L  M  G  T  Y  S  P  S  F  I  S  P  A  T  S  E  S  N  Y  F

       910        920        930        940        950        960        970        980        990
TCAGTGTCAGTCTGCAACATGAACAGCCTGGGAGGGACTCATAATTTGCAACACTCTGAGTCTGATCTCACAGACCTTATTTCAGCCACC
 S  V  S  V  C  N  M  N  S  L  G  G  T  H  N  L  Q  H  S  E  S  D  L  T  D  L  I  S  A  T

      1000       1010       1020       1030       1040       1050       1060       1070       1080
AACTCTCCAATTAGAGGTTTGGACTTCTCAATTGACCCTGCGGAGCTAGATCCAAATTTTAATTTTACTAGATCAGGGTTCTTCACATGA
 N  S  P  I  R  G  L  D  F  S  I  D  P  A  E  L  D  P  N  F  N  F  T  R  S  G  F  F  T  *
```

Figure.S1 Cloning of CtWRKY53 gene

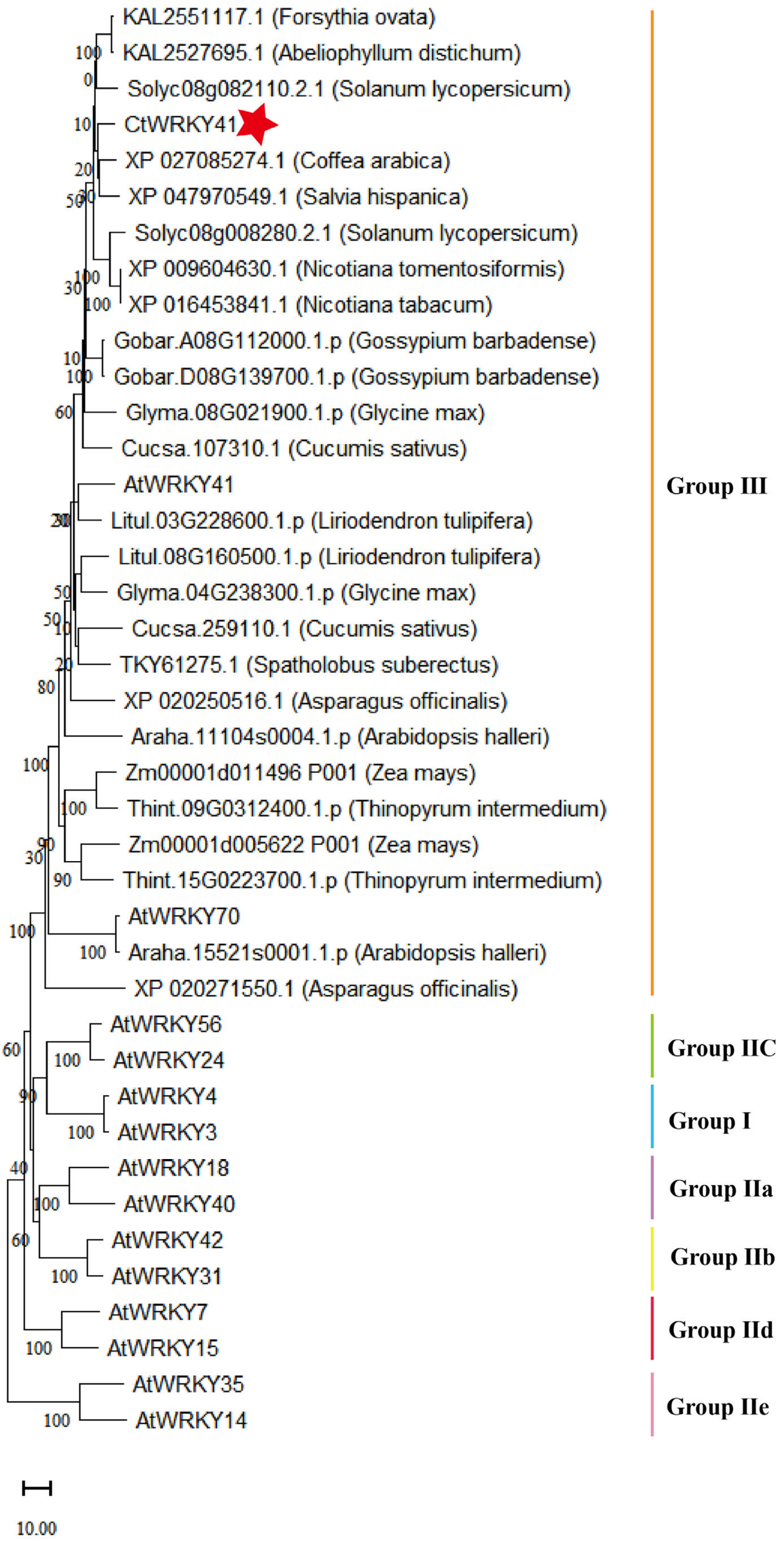

KAL2551117.1 (Forsythia ovata)
KAL2527695.1 (Abeliophyllum distichum)
Solyc08g082110.2.1 (Solanum lycopersicum)
CtWRKY41
XP 027085274.1 (Coffea arabica)
XP 047970549.1 (Salvia hispanica)
Solyc08g008280.2.1 (Solanum lycopersicum)
XP 009604630.1 (Nicotiana tomentosiformis)
XP 016453841.1 (Nicotiana tabacum)
Gobar.A08G112000.1.p (Gossypium barbadense)
Gobar.D08G139700.1.p (Gossypium barbadense)
Glyma.08G021900.1.p (Glycine max)
Cucsa.107310.1 (Cucumis sativus)
AtWRKY41
Litul.03G228600.1.p (Liriodendron tulipifera)
Litul.08G160500.1.p (Liriodendron tulipifera)
Glyma.04G238300.1.p (Glycine max)
Cucsa.259110.1 (Cucumis sativus)
TKY61275.1 (Spatholobus suberectus)
XP 020250516.1 (Asparagus officinalis)
Araha.11104s0004.1.p (Arabidopsis halleri)
Zm00001d011496 P001 (Zea mays)
Thint.09G0312400.1.p (Thinopyrum intermedium)
Zm00001d005622 P001 (Zea mays)
Thint.15G0223700.1.p (Thinopyrum intermedium)
AtWRKY70
Araha.15521s0001.1.p (Arabidopsis halleri)
XP 020271550.1 (Asparagus officinalis)
AtWRKY56
AtWRKY24
AtWRKY4
AtWRKY3
AtWRKY18
AtWRKY40
AtWRKY42
AtWRKY31
AtWRKY7
AtWRKY15
AtWRKY35
AtWRKY14
Group III
Group IIC
Group I
Group IIa
Group IIb
Group IId
Group IIe
10.00

Figure.S2 Phylogenetic relationship between CtWRKY41 and WRKY proteins from other plants. The portion highlighted with a pentacle is CtWRKY41

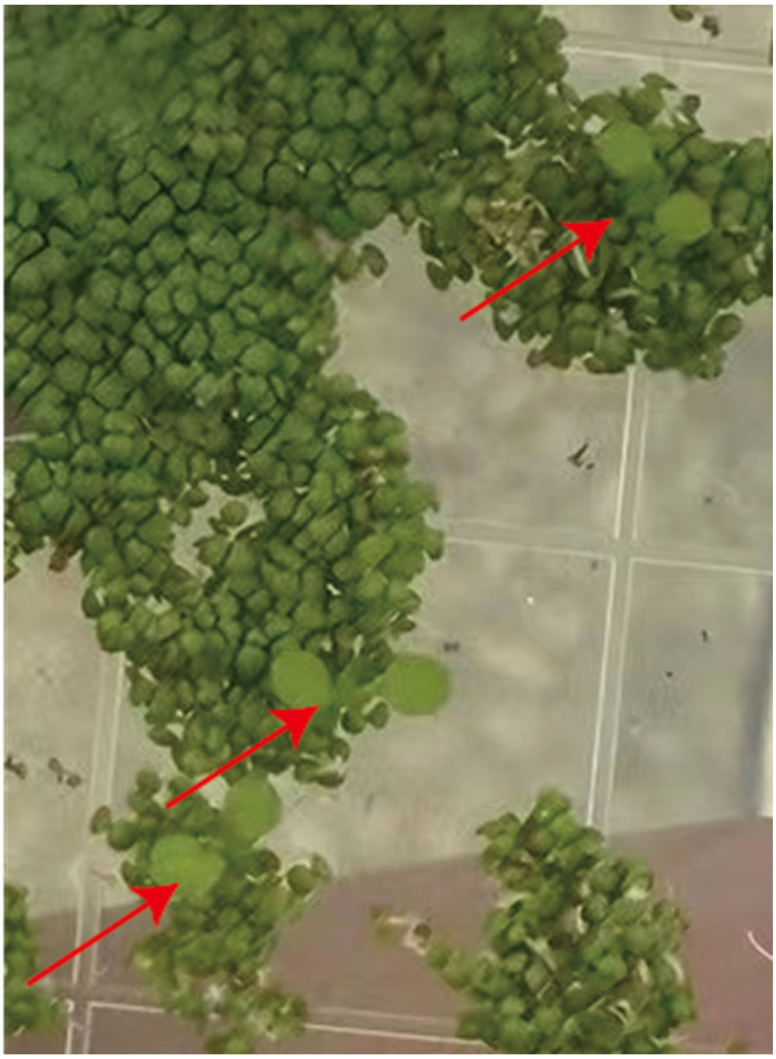

Figure.S3 Screening of transgenic Arabidopsis-positive plants. The red arrow indicates the positive transgenic line.

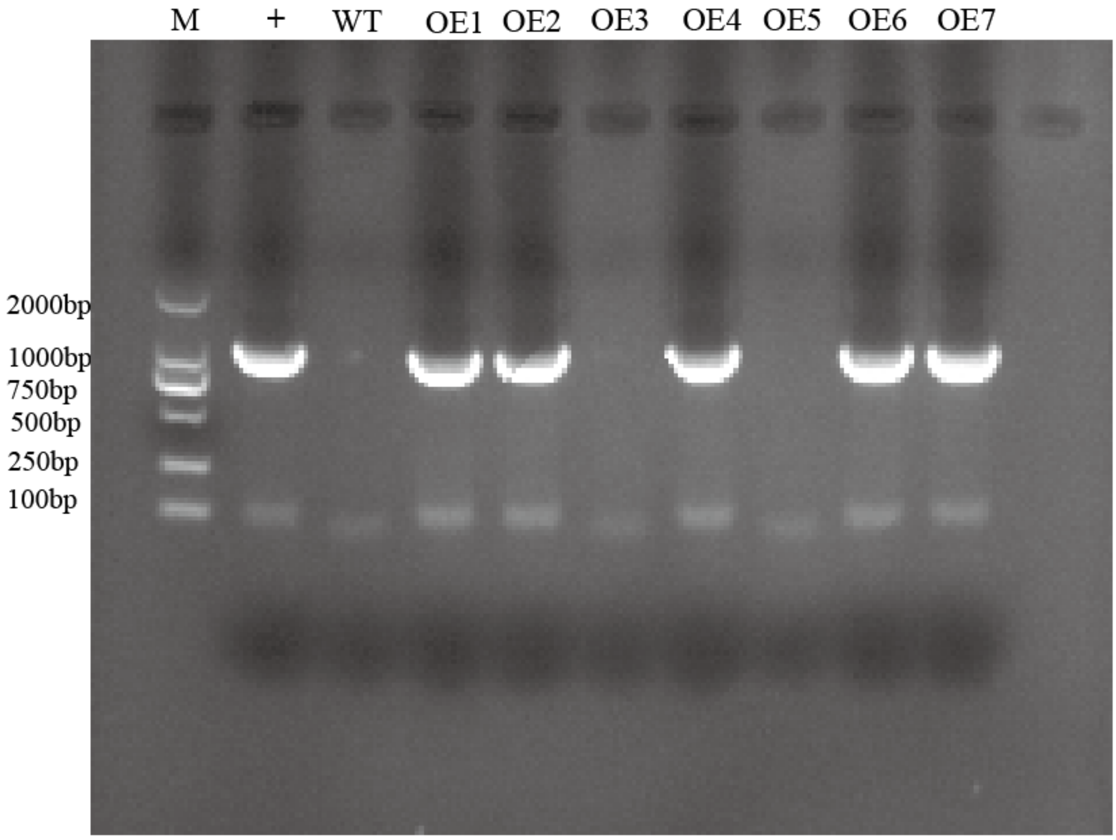

Figure.S4 Note: M: DNA ladder marker 2000; +: PCR amplification of CtWRKY41 in Cynanchum thesioides; OE1-7: CtWRKY41 transgenic lines; WT: Wild-type A. thaliana.

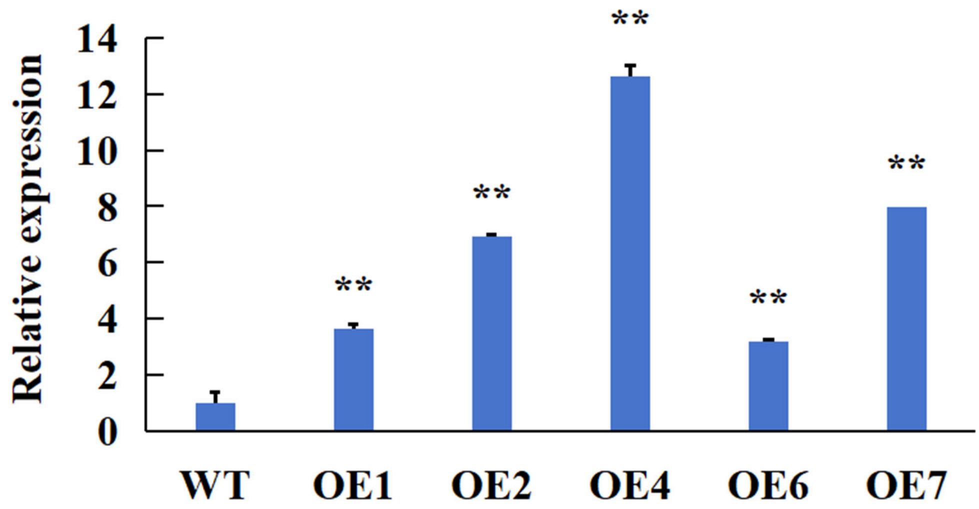

Figure.S5 CtWRKY41 expression levels of wild type (WT), and transgenic lines (OE1, OE2, OE4, OE6, and OE7).

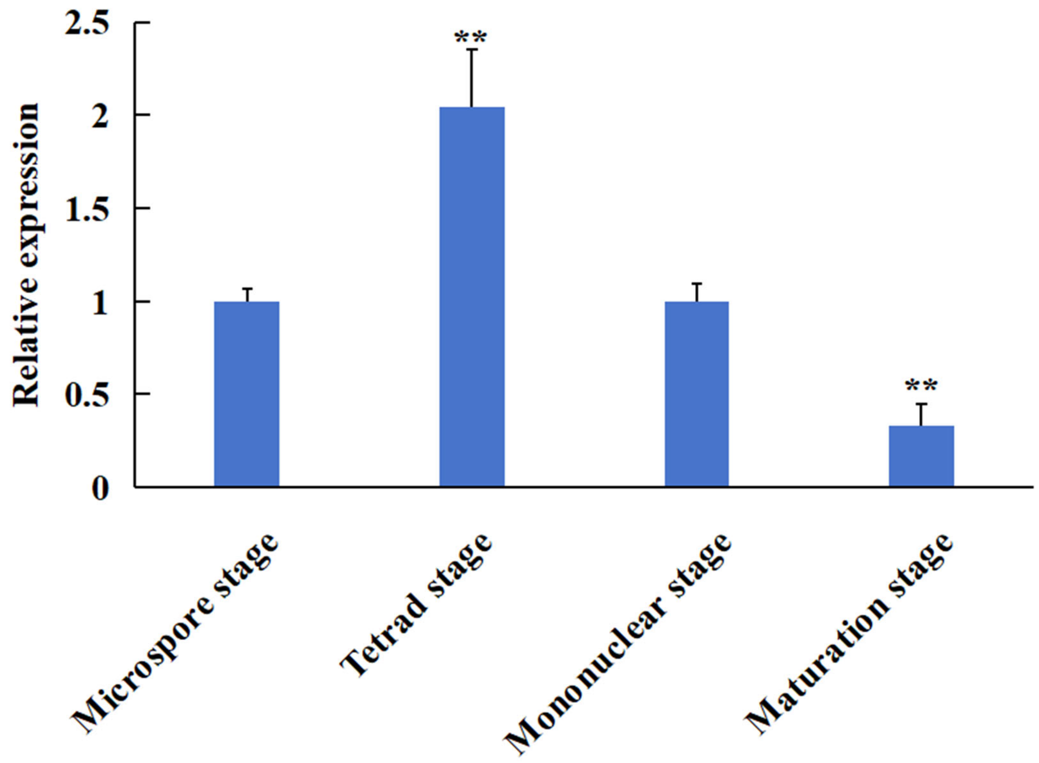

Figure.S6 *CtWRKY41* genes express at different developmental stages of the anther

Table S1 Primers used in this study

Primer	Primer sequences (5'→3')
AtPOD1-F	GCACATACGATAGGAGTCACACA
AtPOD1-R	GACAAGCAACACGCAAGAAC
AtCAT1-F	TCCTGTTATCGTTCGTTTCTCA
AtCAT1-R	CAAAGTTCCCCTCTCTGGTGTA
AtAPX1-F	AAATACGCTGCTGATGAAGATG
AtAPX1-R	GGAGACACACACACACACACAG
AtRD22-F	ATAATCTTTTGACTTTCGATTTTACCG
AtRD22-R	CTTGGACGTTGGTACTTTTCTCG
AtNCED3-F	CAGCTTGTAGCTTTTGGGCTGTA
AtNCED3-R	TAACAGAAACCAGCTGAGCTCGA
AtABI1-F	AGAGTGTGCCTTTGTATGGTTTTA
AtABI1-R	CATCCTCTCTCTACAATAGTTCGCT
AtABI2-R	GATGGAAGATTCTGTCTCAACGATT
At ABI2-R	GTTTCTCCTTCACTATCTCCTCCG
AtCO-F	CACAGGTGAATACAGTCAACACC
AtCO-R	CCATGGATGAAATGTATGCGTTATGG
AtGA20OX-F	CGGTTTTGCGACGACATGAG
AtGA20OX-R	TAGCCCCAGAAGCTCCATGA
AtSOC1-F	GATCGAGTCAGCACCAAACC
AtSOC1-R	TCCTATGCCTTCTCCCAAGA
AtFLC-F	AGCCAAGAAGACCGAACTCA
AtFLC-R	AGCTTCTGCTCCCACATGAT
AtFT-F	TACGAAAATCCAAGTCCCACTG
AtFT-R	AAACTCGCGAGTGTTGAAGTTC
AtActin1-F	CTCCTTTGTTGCTGTTGACTAC
AtActin1-R	GCACAATGTTACCGTACAGATC
CtWRKY41-qPCR F	GGGTTCATCACATTTGTTCCTTCC
CtWRKY41-qPCR R	GCTGCTTAGCTTTCTCCATTCCTT
ACT7-F	AATGAGAGGTTCCGTTGCCC
ACT7-R	GTTGAACCACCACTGAGCAC
CtWRKY41-BDF	atggccatggaggccgaattcATGAATTGTGCTACTAGCTGGGAA
CtWRKY41-BDR	ccgctgcaggtcgacggatccTCATGTGAAGAACCCTGATCTAGTAAA
CtWRKY41-eGFP-F	acgggggacgagctcggtaccATGAATTGTGCTACTAGCTGGGAA
CtWRKY41-eGFP-R	tggcgcgccgggccctctagaTCATGTGAAGAACCCTGATCTAGTAAA
pYES2- CtWRKY41-F	cttggtaccgagctcggatccATGAATTGTGCTACTAGCTGGGAA
pYES2- CtWRKY41-R	tgatggatatctgcagaattcTCATGTGAAGAACCCTGATCTAGTAAA
